# The Progress of Surgery in the Last Twenty-Five Years

**Published:** 1895-09

**Authors:** Horatio Percy Symonds

**Affiliations:** Surgeon to the Radcliffe Infirmary, Oxford.


THE BRISTOL

### Medico-Chirurgical Journal.

SEPTEMBER, 1895.

### THE PROGRESS OF SURGERY IN THE LAST TWENTY-FIVE YEARS.

The Presidential Address, delivered on July 12th, 1895, at the Annual Meeting of the

Oxfordshire Branch of the British Medical Association,

BY

Horatio Percy Symonds.

*Surgeon to the Radcliffe Infirmary, Oxford.*

It is a well-known fact that objects viewed from a distance seem of smaller dimensions than they really are, and so it is with events which are to happen in the far-off future. If a task which might appear insuperable to-day is not to be done for many months, it is regarded as of ready accomplishment until the time for its performance draws near. This is how matters appeared to me when I was asked if I would undertake the office of President of this Branch for the present year. I at once accepted the proffered honour, as if it were an easy duty and one of light importance; but as time went on and the event came within measurable distance, many difficulties presented themselves which I had not foreseen, not the least of these being the fact that I should have to deliver an address to so many whom I

am exceedingly pleased and honoured to welcome to-day as my guests.

With regard to the subject of my address, I wish to say something to you of the progress of surgery which has occurred in the last twenty-five years, during which time I have watched it with continuous pleasure and interest. Never in the last two thousand years has surgery progressed so rapidly; and this is not due simply to gradual general all-round improvements, but entirely to the influence of one man, whose name will be handed down, and referred to as we now refer to Harvey and Hunter. That man is, of course, Sir Joseph Lister, and it is to his work and its results that I wish to ask you to look for a little.

When Lister commenced his work the occurrence of septicæmia, pyæmia, erysipelas, and hospital gangrene were constant factors with which the surgeon had to reckon. Such complications were always recurring in his cases, and the fear of their occurrence with fatal result prevented him from attempting many operations altogether. If in the light of our present knowledge we look back at surgical procedure then, we cannot be surprised that the so-called septic diseases did prevail. I can remember when our preparation of ligatures at the Radcliffe Infirmary was something as follows: Six yards of whitey-brown thread were stretched across the room, and rubbed well with an old piece of beeswax. The thread was then cut in eighteen inch lengths, and attached with pins to the dirtiest possible pincushion, from which, when beaten, dust rose in clouds. This cushion was soiled with blood-clot and discharges from many previous operations. An old surgeon quoted by Lunn said: "I like a good stink in a wound. Give me a wound that is fœtid. I don't care for it a bit; it is wholesome." These merely show what were common characters in the preparations for operation and in the results obtained. It is no wonder that the latter were bad. Lister, dissatisfied with what he found and seeking for the cause, put to himself this question: "How is it that an external wound leads to such grave results in cases of fracture, while cases of simple fracture heal without any trouble? Is it not probably by the atmosphere inducing decomposition of the blood which is effused in greater or less amount around the fragments and among the interstices of the tissues, and to this blood, losing by putrefaction its natural bland character and assuming the properties of an acrid irritant, occasioning both local and general disturbance?"

Now, how does the atmosphere produce decomposition of organic substances? We find that a flood of light was thrown upon this most important subject by the philosophic researches of Pasteur, who demonstrated by thoroughly convincing evidence that it is not to its oxygen or to any of its gaseous constituents that the air owes these hurtful properties, but wholly to minute living particles suspended in it. Applying these principles to the treatment of compound fractures and bearing in mind that it is from the vitality of the atmospheric particles that all the mischief arises, Lister then argued that all that is requisite is to dress the wound with some material capable of killing these septic germs, provided that any substance can be found trustworthy for this purpose, and yet not too potent as a caustic. Lister says that in the year 1864 he was much struck with an account of the remarkable effects produced by carbolic acid upon the sewage of the town of Carlisle, the admixture of a very small proportion not only preventing all odour from the lands irrigated with the refuse material, but, as it was stated, destroying the entozoa which usually infest cattle fed upon such pastures. Lister's first application of his theory was made in Glasgow Royal Infirmary in 1865, the method at that time being to apply lint dipped in pure liquid carbolic acid upon the wound. The first case was unsuccessful, in consequence of improper management, but subsequent trials more than realised his most sanguine anticipations. Now, from that time to this these principles have gradually grown, till now it is universally recognised that "suppuration occurring in a wound made by a surgeon through unbroken skin is due to some oversight on his part." The actual use of carbolic acid to wounds was, as you know, not original to Lister, but was used first by Lemaire, who used it indiscriminately for all diseases, both internally and externally; and Guthrie, in the Peninsula War, used chloride of zinc to all the sloughing wounds with markedly good effect.

To the Listerian surgery there have, of course, been many oppositions, and yet it has gained more and more disciples until, at the present time, we hear of absolutely no opposition, every surgeon now endeavouring to obtain the results which Lister showed could be obtained if his preventive measures were adopted. Those who have opposed Lister ought to be thanked very much by those who have practised his principles with enthusiasm, as it has made the latter all the more careful to carry out the details with still greater accuracy, and their opposition has also called attention to any fallacies existing in the antiseptic proceedings. There have also been many and frequent alterations in the *technique* of antiseptic procedures, and nothing indicates the true greatness of its author more than the way in which he has always recognised defects in his own system, and striven to improve it. He would never be satisfied until he had attained as nearly as possible to perfection. In no detail has this been better illustrated than in the attitude he took up with regard to the use of the spray. Lister had been led by Pasteur's discovery of putrefactive organisms in the air to introduce the carbolic spray in order to kill these organisms, and this spray, being a very obvious instrument, naturally attracted attention; and as the scientific knowledge on the subject advanced, it became more and more evident that the organisms which give rise to the particular form of putrefaction called septic disease come not so much from the air, but from the patient's skin, the hands of the operator, and the latter's instruments. When this was clearly made out, Lister at once acknowledged that the spray was not essential. In the *Lancet* of August, 1883, in answer to the question as to whether he had given up the spray, he writes:—"I have not given up the spray, although I certainly regard it as the least important part of our antiseptic arrangements. Whatever other good it may do, it is a very mild form of antiseptic irrigation, and tends to keep the *entourage* of the wound, including the surgeon's hands and instruments, pure; but if I had not a spray-producer at hand, I should not on that account omit other elements of antiseptic treatment."

The chief recent modification of antiseptic surgery has been the development of aseptic surgery. This is no new principle.

The great aim of antiseptic surgery all along has been to prevent the access of germs to a wound. In aseptic surgery, instead of employing chemical antiseptic solutions to kill the organisms, heat is employed. The advantage claimed is that no irritating chemical material is carried into the wound by instruments, &c.

This aseptic surgery is an addition to antiseptic surgery, for it is quite impossible for any surgeon to do without some form of chemical antiseptic in addition to heat; for it is quite impossible to boil the surgeon's hands or the patient's skin, which are the most dangerous carriers of septic materials into the wounds. A surgeon never knows whether he may not have to arrest hemorrhage in a wound already septic before he is called to a case. Kocher, who has been held up as the great advocate of aseptic surgery, says in his recent book on *Operative Surgery:* "Thanks to antiseptics, we are now able to bring about rapid healing in sutured operation wounds, however large, and the result has been that operative surgery has made extraordinary strides. As soon as we are sure of our antiseptics we may cut down upon any part of the body, not only with a therapeutic, but also with a diagnostic, intent. It is all the more our duty, therefore, in view of the extraordinary widening of the indications for operative treatment, to make our *technique* as perfect as possible." To carry out the sterilisation of the instruments and dressings, we may therefore either use antiseptic solutions or sterilise by heat. As regards sterilisation of the wound, it may be shortly stated that if, as Zimmermann has proved, it is not possible by contact with 1—1000 sublimate solution to sterilise with certainty pieces of flesh which have been momentarily contaminated with micro-organisms, then there is no hope of doing it in wounds. Nevertheless, Zimmermann obtained by such disinfection a very distinct difference in degree, inasmuch as far fewer colonies developed, and these took longer to appear in consequence of having been weakened by the antiseptics. Therefore, sterilising of the hands before operation and the washing-out of a wound after operation with sublimate solution 1—500 is to be advised. Aseptic surgery really consists of the scientific principle of cleanliness, using the word "clean" in the sense of "free from germs." Now, a very common criticism of modern surgery is that it is nothing more than cleanliness, using the word in the ordinary æsthetic sense. This is not the case, and I think if we look back to the pre-antiseptic era we shall find that the surgeon considered apparent cleanliness as most important. An example of this is found in the manner in which Liston treated his amputations in the year 1842. He kept clean lint soaked in cold water between the flaps for two days, and then brought the flaps together with sutures and isinglass plaster, "instead," as he says, "of using resinous plaster, which is a dirty application. When suppuration is fairly established, tepid water and a stimulating lotion are used." You see from this that while cleanliness was considered most important, there was no attempt at primary union, which is the great criterion of success under the Listerian system.

Even in its early developments the antiseptic system showed a decided improvement over results obtained in the immediately preceding epoch. Thus the results of primary amputations had been exceedingly bad, and where secondary amputation was resorted to the results were still more disastrous. In the Franco-Prussian War only a few recoveries took place under such circumstances. In one year, at Munich, 11 out of 17 amputations died. Comparing the statistics of amputation in the pre-antiseptic period with later results, out of 377 cases 110 died. In the cases treated antiseptically, out of 321 cases only 14 died; and if you remove the cases that died of shock the results are still more remarkable, as in the first series six died from shock and in the last eight died from shock. It may be said that the delays of antiseptic procedure add to the shock, but I think that with increased probability ot recovery, more severe cases are submitted to operations if they can be treated antiseptically. In Lister's own wards, before the antiseptic procedures were introduced, out of a series of 35 amputations 16 deaths occurred. The introduction of his system at once led to a change, for out of the next 40 amputations only 6 died. In Volkmann's *clinique* the mortality of the compound fractures before the antiseptic era was very large, for out of 14 such cases 12 died, while when antisepsis was practised, out of 75 compound fractures no death occurred; and in more modern times, over larger groups of cases, the results are even more striking. Thus in 1892, in St. Thomas's Hospital, London, out of 1,108 operations performed, there occurred only 11 deaths from pyæmia—contracted either before or after admission—or about 1 per cent. In 1893, 1,250 operations were performed with 16 deaths from pyæmia, or 1.2 per cent. In the Edinburgh Infirmary during 1893, 2,670 operations were performed, and 18 deaths from pyæmia occurred, or only .7 per cent. Not only, however, has the practice of antisepsis decreased the actual mortality, but the time of healing of wounds has been strikingly diminished. In former days an excision of the breast averaged from three to six months in healing, and for any ordinary accidental wound nine weeks were allowed for the patient to remain in hospital. At the present time fourteen days is the average time for an excision of the breast to heal, and many minor wounds which were formerly admitted into hospitals are now treated as out-patients. The saving of suffering to patients which has come about as a consequence of the introduction of antiseptics is thus beyond all calculation.

But mere statistics do not show all the advantages which have come from the Listerian system. One great change that has come over surgery is the removal of formerly-admitted anatomical restrictions upon surgical operations. The operation of trephining the skull no doubt dates from prehistoric times, but interference by the surgeon with the brain itself has only been done of late years. The history of trephining is interesting. The ancients trephined in all fractures, either to relieve symptoms if they were present, or to prevent them coming on if they were absent. They trepanned along the whole course of the fracture, so as to saw it out. Godifredus, chief surgeon to the States of Holland, mentions with particular exultation the performance of this operation by a friend, who trepanned the cranium of the Count of Nassau twenty-seven times; and that the fact might be established on indisputable authority, he made the said Count of Nassau, after he was recovered, write the following curious certificate on the 12th day of August, 1664: "I, the under-written Philip, Count of Nassau, hereby declare and testify that

Mr. Henry Chadborn did trepan me in the skull twenty-seven times, and after that did cure me well and soundly." Regarding such procedures, Pirrie wrote in 1840: "These fractures and the numerous inventions of instruments for cutting the skull are sad monuments of the surgery of past ages." Desault, after that time, in Paris, pointed out the numerous unfortunate results of the operation, and stated that its practice was reduced down to only trephining in compound depressed fractures of the skull, and in depressed fractures with symptoms. It is interesting now to see Macewen's statistics and Victor Horsley operating on the skull with a trephine with two-inch diameter worked by an electro-motor.

Macewen, in his famous address on cerebral surgery before the British Medical Association in Glasgow in 1888, pointed out that two factors were necessary for the introduction of modern cerebral surgery. It was necessary (1) to adopt means whereby immunity from the inflammations which so constantly attended brain-lesions could be secured; and (2) to endeavour to gain a better physiological knowledge, in the hope that light might be shed by the localisation of cerebral lesions. He further says: "Experience gained by me showed that not only compound fractures of the skull, but large osseous defects in the cranial vault accompanied by extensive lesions of cerebral substance were quite amenable to treatment, exhibiting no tendency to inflammatory action as long as the tissues were preserved aseptic." Macewen's record bore out with what success he carried out his principles—out of twenty-one cases only three deaths resulted. His successes included abscess of the brain, tumour of the dura mater, intracranial subdural effusion, syphilitic tumour, cysts of the brain—all of them conditions which formerly surgery would never have thought of attacking.

Then, again, radical cure of hernia by open wound was performed by Celsus, but fell entirely into disuse until the year 1875, and now every organ of the body has been treated by the surgeon. The field of direct surgical interference was formerly limited to the limbs, and to the common coverings of the great serous membranes. Surgeons were content to feel that the peritoneum, the kidney, and the lung were beyond the limits of legitimate surgical interference.

Perhaps the most obvious of the advances in operative surgery have been in abdominal surgery; although some surgeons have asserted that they have abandoned Listerism, forgetting that Listerism is a treatment of wound-surroundings more than the treatment of the wounds. The statistics of ovariotomy are remarkable. Dr. Clay's statistics, published in 1871, were two hundred and twelve recoveries to one hundred and eighty-seven deaths. Knowsley Thornton, in 1890, gives his mortality—under the antiseptic system—at two per cent. The success also of Cæsarian section at the present time is better than it was ever expected to be even fifteen years ago, and now Porro's operation is being discarded for the less serious operation of suturing the uterus after the operation. Again, craniotomy is being discarded for the modern Cæsarian section; surgeons being encouraged by such success as that of Cameron, who had twenty-two consecutive cases of the operation without a death. Our surgery, too, to-day is limited not so much by the delicate anatomical structures and relations of a particular organ, as by the physiological work which it has to perform. Thus, we cannot operate upon a kidney when we suspect the opposite one to be diseased; and again, although tumours may be removed from the surface of the brain, yet such parts as the medulla and central portions of the brain must not be interfered with. Here it may be noted that there are some organs which can be removed more easily when their anatomical relations are abnormal; for instance, movable spleen and a prolapsed uterus. This latter operation can be performed easily by the abdominal method; whereas if it be attempted upon the normal cadaver, it is impossible to leave the stump intact. So again the operation for removal of the thyroid gland is much more easily accomplished when the gland is of large size and its anatomical relations to a certain extent altered. To remove a normal thyroid gland would be much more difficult. Although many operations are now performed which were never thought of fifteen years ago, yet amputation of limbs is much less frequently employed now than it was formerly. We excise joints that are diseased in preference to amputating the limb, and this is only possible in consequence of the introduction of antiseptics. Thus Saxtorph found that the mortality after excision sank from 33 per cent. to 3 per cent. as a consequence of the practice of antiseptic surgery. Again, many compound fractures are now treated by conservative measures where formerly amputation was the only possibility of saving the life of the patient antiseptically. Further, osteotomy for deformity has been proved as safe as subcutaneous tenotomy.

One of the most striking changes in surgical treatment is that of tuberculous abscesses, empyema, psoas abscess, and the like. The old method of incision and drainage, with more or less imperfect washing out of the abscess cavity, was a grim failure. It often led to rapid death from septicæmia or pyæmia; and when it did not, the suppuration continued and the abscess was said to degenerate into a sinus. So bad were the results that many of the most experienced surgeons refused to operate at all, and left the abscess to burst; for they recognised that where they could not interfere with advantage, it was at least their duty not to hasten their patient's death by an operation. So in a less formidable malady—namely, tuberculous glands—the old method only slightly anticipated Nature; whereas now, once the disease is recognised, the whole of the glands suspected to contain the tubercle bacilli are removed.

Perhaps another of the more recent improvements in surgery is the operation of suprapubic lithotomy, which now entirely supersedes any other operation in the adult; and I think, even in children, it will ultimately be thought the best operation, on account of not injuring important organs at the neck of the bladder. The result to be wished for, and which the surgeon should endeavour to obtain, is to remove the calculus with as small an incision into the bladder as possible, afterwards to suture the viscus with buried catgut and close the abdominal wound in the usual manner, the after-treatment and recovery of the patient being perfectly straightforward.

Having mentioned catgut to be used in this manner, a glance on passing at the history of the use of catgut may be interesting. Galen, Ambrose Paré, and Sir Astley Cooper all used the catgut ligature; but it was not until antiseptic surgery was firmly established that catgut could be used as an organising ligature and also as a buried suture, for in a septic wound it simply acts as an irritant and the wound does not heal till it has ulcerated out. Those who have commenced surgical work during the last twenty years have no idea of the extent of our gain in this respect. They may partly imagine, but cannot realise, the want of such an aid as a suture which can be used for bringing together the deep structures in a wound and closing the wound over them. Just to give an instance of its use in the oldest of operations—say, amputation of the thigh in the case of a child or delicate young adult, for disease where the periosteum, with the muscles attached, tends to strip off the bone like a glove off a finger—what trouble often arose in keeping the end of bone covered! Now we can rectify this by putting stitches into the periosteum and drawing them together over the bone, or by the purse-string suture. Silk, of course, can be used, but then it has to become isolated, it is never organised, and the knot frequently becomes a mechanical irritant. Silk in the abdominal cavity is quite satisfactory, on account of the ample room there is for its isolation.

With regard to those cases that are already suffering from erysipelas, septicæmia, pyæmia, or puerperal fever when first seen by the surgeon, up till now all that could be done was to maintain the strength of the patient and allow the disease to be fought out. But now we seem able to hope that not only can we prevent septic disease occurring, but that we shall be able to cure it when it does occur. Since this paper was commenced, there has been a communication sent to the *Surgical Review* of Paris describing an antistreptococcic serum capable of arresting the numerous forms of infection caused by streptococci. About three years ago it was shown by Roger that cultures of streptococci contain two antagonistic substances, one of which diminishes, whilst the other increases, the resistance of inoculated animals. The former is destroyed by heat, so that with a culture raised to a temperature of 230° F. animals can be vaccinated against streptococcic infection. The serum of animals thus treated acquires the property, not of destroying, but of attenuating, the microbes which are introduced into them, and of checking infection set up by virulent cultures. Serum taken from a mule which had been extensively injected with sterilised cultures was used on a woman suffering from puerperal fever. After unsuccessful injections of 8 and 16 c.cm., a third injection of 25 c.cm. on the third day was followed by defervescence and rapid recovery. Three other cases of recovery have been reported by Charrin and Roger at the Biological Society of France. The prospects of success in this direction are favoured by the fact that Marmorek, another and independent investigator, has recently recorded a series of 45 cases of erysipelas in which the patients rapidly recovered after the injection of specially-prepared serum. Jacquot has since this communicated the case of a woman attacked by puerperal septicæmia in which intra-uterine injections and quinine were without effect. He then injected Roger and Charrin's antistreptococcic serum. After three injections of the serum the patient seemed well.

I cannot close this poor attempt to indicate the main course of the advance of surgery in the last twenty-five years without once more reiterating that it is to one master-mind that we owe the greatest impulse that surgery has ever felt. We cannot help feeling a special pride that the name that shines with an unrivalled splendour on the page of surgical history is that of the Englishman, Joseph Lister; and although this is not a University gathering, it is well to remember that Harvey was Warden of Merton College, Hunter commenced his education at St. Mary's Hall in Oxford University, and that Pasteur and Lister have received the honorary degree of D.C.L. in the University of Oxford.

---

On his retirement from King's College Hospital, Sir Joseph Lister was presented on July 31st with his portrait. Sir John Erichsen, in making the presentation, referred to the enormous progress which had been made in surgery, mainly owing to the introduction of antiseptic surgery, a boon which had made Sir Joseph Lister one of the greatest benefactors of mankind. Sir Joseph, who was very warmly received, sketched the steps which had led him to enunciate the theory of the antiseptic treatment of wounds. It had been to him one of the chief joys of life to impart to students the principle of the noble and beneficent art of surgery, and he was extremely gratified by the testimonial which had been presented to him that day by past colleagues and pupils, proving, as it did, that his efforts had not been altogether in vain.

A Committee has been formed to present a portrait of Sir Joseph Lister to the Royal College of Surgeons of England.

